# *Agaricus brasiliensis* Mushroom Protects Against Sepsis by Alleviating Oxidative and Inflammatory Response

**DOI:** 10.3389/fimmu.2020.01238

**Published:** 2020-07-01

**Authors:** Kely Campos Navegantes-Lima, Valter Vinicius Silva Monteiro, Silvia Leticia de França Gaspar, Ana Ligia de Brito Oliveira, Juliana Pinheiro de Oliveira, Jordano Ferreira Reis, Rafaelli de Souza Gomes, Caroline Azulay Rodrigues, Herta Stutz, Vanessa Sovrani, Alessandra Peres, Pedro Roosevelt Torres Romão, Marta Chagas Monteiro

**Affiliations:** ^1^Neuroscience and Cellular Biology Post Graduation Program, Institute of Biological Sciences, Federal University of Pará, Pará, Brazil; ^2^Center for Research in Inflammatory Diseases (CRID), Department of Pharmacology, Ribeirão Preto Medical School, University of São Paulo, São Paulo, Brazil; ^3^Graduate Program in Basic and Applied Immunology, Ribeirao Preto Medical School, University of São Paulo, São Paulo, Brazil; ^4^School of Pharmacy, Health Science Institute, Federal University of Pará, Pará, Brazil; ^5^Pharmaceutical Science Post-Graduation Program, Faculty of Pharmacy, Federal University of Pará, Pará, Brazil; ^6^Department of Food Engineering, Midwest State University-UNICENTRO, Guarapuava, Brazil; ^7^Department of Biochemistry, Federal University of Rio Grande de Sul, Porto Alegre, Brazil; ^8^Laboratory of Cellular and Molecular Immunology, Department of Basic Health Sciences, Federal University of Health Sciences of Porto Alegre, Porto Alegre, Brazil

**Keywords:** polymicrobial sepsis, CLP, *Agaricus brasiliensis*, sun mushroom, antioxidant, immunomodulator, protection

## Abstract

Sepsis is characterized by the host's dysregulated immune response to an infection followed by a potentially fatal organ dysfunction. Although there have been some advances in the treatment of sepsis, mainly focused on broad-spectrum antibiotics, mortality rates remain high, urging for the search of new therapies. Oxidative stress is one of the main features of septic patients, so antioxidants can be a good alternative treatment. *Agaricus brasiliensis* is a nutraceutical rich in bioactive compounds such as polyphenols and polysaccharides, exhibiting antioxidant, antitumor, and immunomodulatory activities. Here, we investigated the immunomodulatory and antioxidant effects of *A. brasilensis* aqueous extract in the cecal ligation and puncture (CLP) sepsis model. Our data showed that aqueous extract of *A. brasiliensis* reduced systemic inflammatory response and improved bacteria clearance and mice survival. In addition, *A brasiliensis* decreased the oxidative stress markers in serum, peritoneal cavity, heart and liver of septic animals, as well as ROS production (*in vitro* and *in vivo*) and *tert*-Butyl hydroperoxide-induced DNA damage in peripheral blood mononuclear cells from healthy donors *in vitro*. In conclusion, the aqueous extract of *A. brasiliensis* was able to increase the survival of septic animals by a mechanism involving immunomodulatory and antioxidant protective effects.

## Introduction

According to the World Health Organization (WHO), sepsis leads to about 6 million deaths per year worldwide (1) and is considered the main cause of death in intensive care units, especially in patients with comorbidities (2, 3). Sepsis is currently defined as a syndrome caused by a dysregulated immune response to infection (4) accompanied by an imbalance between pro-oxidant and antioxidant defenses in response to pro-inflammatory cytokines, nitric oxide (NO) and reactive oxygen species (ROS), which can cause lipid peroxidation, DNA damage, mitochondrial dysfunction, and multiple organ failure (5–7). Thus, new therapies based on compounds with antioxidant and/or immunomodulatory action may be effective as alternative therapy.

To study the pathogenesis and therapeutic targets in sepsis, several animal models have been widely used, but cecal ligation puncture (CLP) procedure is considered one that most closely resembles human sepsis (8). Recently, our group showed that in a murine model of moderate CLP-induced sepsis, animal mortality (up to six days after sepsis) was correlated with increased leukocyte migration to the peritoneal cavity and oxidative stress in several organs (spleen, heart, liver and lung) within 24 h of infection (9) and that the pretreatment of animals with salivary gland extract from *Aedes aegypti* improved mice survival through immunomodulatory and antimicrobial effects associated with lower oxidative status (decreased lipid peroxidation and increased antioxidant defense) (9). In this regard, it is of great interest to research new therapies with antioxidant and immunomodulatory properties through nutraceuticals such as *Agaricus brasiliensis* (Ab) (10).

Ab is a mushroom rich in bioactive compounds such as organic acids, amino acids, phenolic compounds and polysaccharides such as β-glucans (11, 12). The β-glucans found in mushrooms like Ab have a β-(1–3) structure associated with β-(16), which is able to stimulate cellular and humoral immune response, increase NO production, phagocytosis and lymphocyte proliferation (13–15). According to Carvajal et al., Ab present compounds such as lactic and fumaric acid, as well as secondary metabolites such as sesquiterpenes, steroids, anthraquinones, quinolines and derivatives of benzoic acid, inhibitors of bacterial growth (10). In addition, Ab has a high antioxidant potential mainly due to the presence of phenolic compounds such as gallic acid, serum acid and pyrogallol, karmic acid and other compounds such as ascorbic acid and α-tocopherol (11, 12, 16).

Therefore, considering sepsis as one of the major global public health challenges, the urgency for new therapeutic alternatives and the immunomodulatory properties of Ab, the aim of this study was to evaluate for the first time the effects of prophylactic administration of aqueous extract of Ab on survival, immunological and oxidative parameters in a murine sepsis model.

## Materials and Methods

### Ethics Statement

This study was carried out in strict accordance with the recommendations of the Guide for the Care and Use of Laboratory Animals of the Brazilian National Council of Animal Experimentation (http://www.sbcal.org.br/) and the NIH Guidelines for the Care and Use of Laboratory Animals. The institutional Committee for Animal Ethics of Federal University of Pará/UFPA (CEUA, Protocol: 02/15) approved all the procedures used in this study.

To *in vitro* tests, human venous blood was collected from healthy volunteers that signed the Informed Consent Form (ICF). This study was approved by the Institutional Committee of Ethics in Research involving human beings from the health sciences sector of UFPA (CEP-ICS/UFPA), under n° 3544380 and CAAE 12776619.0.0000.0018.

### Mice

Male Swiss mice (7–8 weeks old) were used in this study and were obtained from the Animal Facility of the Federal University of Pará. Mice were kept in cages under controlled conditions of temperature (22 ± 3°C), light (12 h light/dark cycle) with food and water *ad libitum*, and acclimatized conditions for 3 days before use.

### Preparation of *Agaricus brasiliensis* (Ab) Aqueous Extract

Ab was kindly donated by Dr. Herta Stutz Dalla Santa from the fungi collection of bioprocesses of the Bioprocesses Laboratory, Food Engineering Department, Universidade Estadual do Centro Oeste (UNICENTRO), Paraná, Brazil. To obtain an Ab aqueous extract rich in bioactive substances such as carbohydrates, in special β-glucans, proteins and phenolic compounds (17–19), we used a methodology described before (20), where 20 g of dried and pulverized mycelium of Ab were boiled in 20 mL of distilled water for 10 min and then the solution was filtered and lyophilized. A stock solution at 100 mg/mL was prepared in sterile distilled water and used for *in vivo* (135 mg/Kg) and *in vitro* experiments (2.81 and 22.5 mg/mL). These doses were chosen based on *in vitro* tests of cytotoxicity using macrophages and peripheral blood mononuclear cells. Before initiate the experimental sets, the antioxidant activity of Ab aqueous extract was confirmed by the *in vitro* assay for total antioxidant activity (TAC) (data not shown).

### Design of *in vivo* Experiments

**Figure 1:**
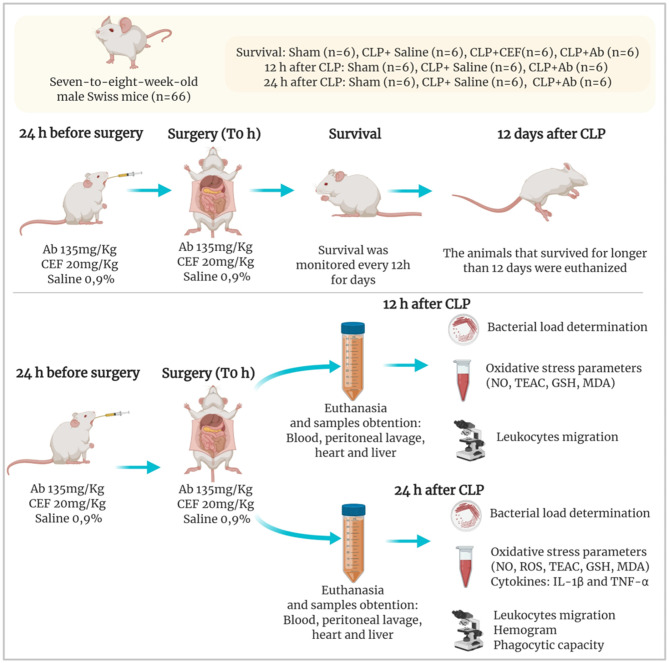
Experimental protocol of CLP model and pre-treatments. Ab, aqueous extract of *Agaricus brasiliensis;* CLP, cecal ligation and puncture; CEF, ceftriaxone; CFU, colony-forming unit; GSH, Glutathione; MDA, malondialdeihyde; ROS, reactive oxygen species; TEAC, Trolox equivalent antioxidant capacity.

The animals were separated into 4 groups according to the treatment schedule, as follows: saline (saline 0.9% + cecal ligation and puncture –CLP/ *n* = 18 animals), Ceftriaxone (Cef −20 mg/kg + CLP/ *n* = 6 animals), aqueous extract of *Ab* (Ab −135 mg/kg + CLP, *n* = 18 animals) and sham (surgery control, *n* = 18 animals). All treatments were administered in a volume of 100 μL orally by gavage, 24 h before and immediately before CLP induction (time 0). In the first set of experiments, 24 animals were used to monitor the survival rate during 12 days. In the next set of experiments 36 animals were used, septic mice were euthanized at specific time points to evaluate the therapeutic potential and immunomodulatory/antioxidant activities of Ab. In this study, 18 mice (6/group) were euthanized 12 h after CLP to analyze oxidative stress parameters and 18 mice (6/group) were euthanized 24 h after CLP to analyze pro-inflammatory cytokines based on previous studies (9, 21) (Figure 1).

#### CLP Model

The polymicrobial sepsis was induced using the cecal ligation and puncture (CLP) model according to D'Acampora and Locks, with some adaptations (9, 22). To summarize, animals were anesthetized with intraperitoneal injection of ketamine (100 mg/kg) and xylazine (10 mg/kg), a small one-centimeter laparotomy performed, and the cecum exposed and then ligated using a 3–0 silk suture. Then, the cecum was punctured one single time with 22G needle to induce a moderate severity CLP. The cecum was gently squeezed to extrude a small amount of fecal content and was left to its original position in the abdominal cavity. Sham-operated mice underwent the same procedure, except for ligation and perforation of the cecum. The abdominal wall was closed, and fluid resuscitation was conducted with subcutaneous injection of 1 mL of saline 0.9%.

### Survival and Weight Analysis of Septic Mice

After induction of sepsis, mice of each group (*n* = 6) were weighted twice a day for twelve consecutive days. To reduce suffering, mice presenting signs of imminent death (i.e., ataxia, inability to maintain upright position, tremor, and/or agonal breathing) were euthanized by ketamine/xylazine (>100/10 mg/kg, sc) overdose. The animals that survived for longer than 12 days were euthanized. The survival rate and weight were calculated followed by delineation of survival and weight curve.

### Blood Samples and Leukocyte Counts

Peripheral blood was obtained by cardiac puncture of mice anesthetized with ketamine (100 mg/kg) and xylazine (10 mg/Kg) at 12 and 24 h after CLP induction. Aliquots (500 μL) of blood collected with ethylenediamine tetraacetic acid (EDTA) 5% were analyzed using an automatic hematologic analyzer *(*Hematoclin 2.8 VET, Starlab, Salvador, BA, BRA) and blood samples (1000 μL) collected without anticoagulant were used to obtain serum for analyses of oxidative stress (12 and 24 h post CLP) and cytokines (24 h post CLP).

### Cytokines Measurement

The levels of TNF-α and IL-1β in serum and peritoneal lavage fluid collected at 24 h post CLP induction were quantified by Enzime-Linked Immunosorbent Assay (ELISA) using an appropriate commercial kit (R&D Systems, Minneapolis, Canada) according to the manufacturer's instructions. The detection limits of each cytokine were IL-1β, 12.5–800 pg/mL with sensitivity of 4.8 pg/mL; TNF-α, 10.9–700 pg/mL with sensitivity of 7.21 pg/mL.

### Determination of Nitric Oxide (NO) Production

The nitrite (NO_2_) was estimated colorimetrically at 12 and 24 h post CLP on the basis of reduction of nitrate to nitrite using Griess method (23). Nitrite level was determined in 100 μL of samples (serum and lavage peritoneal) incubated with an equal volume of Griess reagent for 10 min at room temperature. The absorbance was measured at 550 nm and calculated from a standard curve with sodium nitrite expressed per μMol/mL (24).

### Bacterial Load Determination

For determination of colony-forming units (CFU) in blood and peritoneal fluid of mice (*n* = 6 per/group), 10 μL of samples were diluted with sterile Phosphate-Buffered Saline (PBS) 1:10, and then 10 μL of each dilution was cultured in Müller Hinton Agar and incubated at 37 °C for 24 h. The colonies were counted and expressed in CFU/mL.

### Peritoneal Leukocyte Counts

Twenty-four h after sepsis induction, peritoneal cells of animals were harvested with 3 mL of PBS containing 1 mM EDTA and the number of total leukocytes was determined using a hemocytometer. The number of differential cell counts was carried out counting a total of 200 cells on cytocentrifuge slides stained with panoptic dye. The results are presented as the number of neutrophils and mononuclear cells per cavity.

#### Phagocytic Capacity of Peritoneal Macrophages

The phagocytic capacity of peritoneal macrophages of septic and sham mice was evaluated as previously described (25). Peritoneal macrophages from sham, CLP-saline and CLP-Ab groups were collected 24 h post sepsis induction and incubated in 96-well microplates (2 x 10^5^ cells/well) for 40 min at 37°C and 5% CO_2_. Then, 10 μL of neutral-red stained zymosan (1 × 10^8^ particles/mL) were added to each well and after 30 min the supernatants were removed and cells fixed with Baker's formol-calcium (4% formaldehyde, 2% sodium chloride, 1% calcium acetate) for 30 min. Following, the cells were washed two times by centrifugation in PBS (450g for 5 min). After solubilization of neutral-red stain with 0.1 mL of acidified alcohol (10% acetic acid, 40% ethanol in distilled water) the absorbance was measured in a microplate reader at 550 nm.

### Measurement of Reactive Oxygen Species (ROS) Production

Reactive oxygen species (ROS) production in peritoneal macrophages of septic animals was quantified using 2′,7′-Dichlorodihydrofluorescein diacetate (H_2_DCF-DA) (26). Briefly, peritoneal cells obtained 24 h post CLP induction were incubated at 37° C during 15 min with 30 mM N-(2-Hydroxyethyl)piperazine-N′-(2-ethanesulfonic acid) (HEPES) (pH 7.2), 200 mM KCl, 1 mM MgCl_2_, and 16 μM of H_2_DCF-DA. The conversion of DCFH-DA to the fluorescent product DCF was measured using a fluorescence microplate reader (Victor 2, Perkin Elmer) every 5 min during 30 min with excitation/emission at 488/530 nm. Background fluorescence was determined before the addition of H2DCF-DA and data were expressed as fluorescence intensity.

### Determination of Lipid Peroxidation

Lipid peroxidation was measured in serum, peritoneal cavity, heart and liver samples collected from septic animals at 12 and 24 h post CLP induction as an indicator of oxidative stress, using the thiobarbituric acid-reactive substances (TBARS) assay (27, 28). Briefly, samples were mixed with 0.05 M trichloroacetic acid (TCA) and 0.67% thiobarbituric acid (TBA; Sigma-Aldrich, St. Louis, MO) in 2 M sodium sulfate, and heated in a water bath at 94°C for 90 min. The chromogen formed was extracted in n-butanol and measured at 535 nm. An MDA standard solution was used to construct a standard curve against which unknown samples were plotted. Results are expressed as malondialdehyde equivalents in nmol/L.

### Total Evaluation of Trolox Equivalent Antioxidant Capacity (TEAC)

The total antioxidant capacity (TAC) of serum, peritoneal fluid, heart and liver samples of septic mice (collected 24 h post CLP induction) was evaluated by Trolox ((±)-6-Hydroxy-2,5,7,8-tetramethylchromane-2-carboxylic acid; Sigma-Aldrich) equivalent antioxidant capacity assay (TEAC), which provides relevant information that may effectively describe the dynamic equilibrium between pro-oxidant and antioxidant compounds. In this assay, 2,2′-Azino-bis (3-ethylbenzothiazoline-6-sulfonic acid) diammonium salt (ABTS) (Sigma Aldrich) was incubated with potassium persulphate (Sigma Aldrich) to produces ABTS^∙+^, a green/blue chromophore. The inhibition of ABTS^∙+^ formation by antioxidants in the samples were expressed as Trolox equivalents, determined at 740 mm using a calibration curve plotted with different amounts of Trolox (Sigma Aldrich) (29, 30).

### Glutathione (GSH) Levels

The level of GSH was determined in samples of serum and peritoneal lavage fluid of septic mice at 12 and 24 h post CLP induction using Ellman's reagent (31). This assay was based on the production of yellow color when 5,5′-Dithiobis(2-nitrobenzoic acid) (DTNB) is added to compounds containing sulfhydryl groups. The GSH concentration was determined using a standard curve constructed with different concentrations of GSH in the reduced form. The absorbance was recorded at 412 nm in a microplate reader (SpectraMax 250, Molecular Devices, Union City, CA, USA) and results were expressed in μmol/mL.

### *In vitro* Studies

#### Peripheral Blood Mononuclear Cells (PBMC) Isolation and *in vitro* Stimulation

PBMC of healthy volunteers who were abstainers of alcohol and tobacco (both sexes, ages 20 to 45 years) were isolated from blood using Ficoll (Sigma-Aldrich). PBMC viability was determined by trypan blue exclusion and the viability was always >95%. Then, the cells were washed and suspended in Roswell Park Memorial Institute-1640 medium (RPMI-1640, Sigma-Aldrich) supplemented with 2 g/L sodium bicarbonate, 10% fetal bovine serum (FBS, Sigma-Aldrich), 2% glutamine, and 100 U/mL penicillin-0.1 mg/mL streptomycin (Sigma-Aldrich) and incubated *in vitro* with RPMI medium (control), *tert*-Butyl hydroperoxide (tBHP: 200 μM) or tBHP plus *Ab* (22.5 mg/mL) for 30 min at 37°C.

#### Measurement of Reactive Oxygen Species (ROS) Production

In this assay, mice were injected intraperitoneally with 2.5 mL of 3% thioglycollate (Sigma-Aldrich) and 48 h latter, peritoneal macrophages were harvested as previously described in 2.10. Macrophages (2 × 10^5^) were incubated *in vitro* with tBHP (40 μM) or tBHP plus *A. brasilienses* (22.5 mg/mL) for 30 min at 37°C. ROS production was detected as described in item 2.11 (32).

####  DNA Damage Using Comet Assay

PBMC were treated *in vitro* with tBHP (200 μM) or tBHP plus *A. brasilienses* (2.81 or 22.50 mg/mL) for 3 h. To perform the Comet assay, each sample was mixed with low melting-point agarose at 37°C to a final concentration of 0.5%. The mixture (100 μL) was added to the slides precoated with 1.5% normal-melting-point agarose to retain the agarose cell suspension. The drop containing cells was covered with a glass cover slip and left at 4°C for 5 min. The slides were treated with a lysis solution (2.5 M NaCl, 100 mM EDTA, 100 mM TRIS, 1% Triton X-100, 10% DMSO, pH ~ 10.2) for 24 h at 4°C. After, the slides were placed horizontally on an electrophoresis tray and the resultant nucleoids were immersed in electrophoresis buffer (300 mM NaOH, 100 mM EDTA, pH > 13) for 20 min at 4°C to cleave the alkali-labile sites. Then the electrophoresis was started using an electric field of 23 V/cm for 20 min. At the end of the process, the slides were gently removed from the tray and washed with distilled water for 5 min for neutralization. The slides were dehydrated for 3 min in absolute ethanol and were then air dried. Finally, the slides were stained with ethidium bromide (20 μg/mL) and viewed using fluorescence microscopy ZEISS AxioCam HRc with green barrier filter 510–560 nm and 400x coupled to a video camera. The cell images were analyzed using Tritek Comet Score Freeware 1.6 software. Registered parameters included the percent of DNA in the tail (Tail DNA %), Tail Length (TL), Tail Moment (TM), and Olive Moment (OM) as marker of DNA damage. One hundred comets were scored randomly for each concentration employed. All steps described previously were carried out in a darkroom to prevent the interference of additional DNA damage.

### Statistical Analysis

Statistical analyses were performed using Graphpad Prism 6 software (GraphPad Software Inc., La Jolla, USA). We assessed differences in the survival groups after CLP using Kaplan-Meier analysis followed by a log-rank test. Other data were analyzed using Analysis of Variance (ANOVA) followed by Tukey multiple comparison test. Data are presented as mean ± SD values. In all cases the significance level adopted was 5% (*p* < 0.05).

## Results

### Aqueous Extract of *A. brasiliensis* Improve Survival and Inflammatory Systemic Markers in Septic Mice

**Figure 2:**
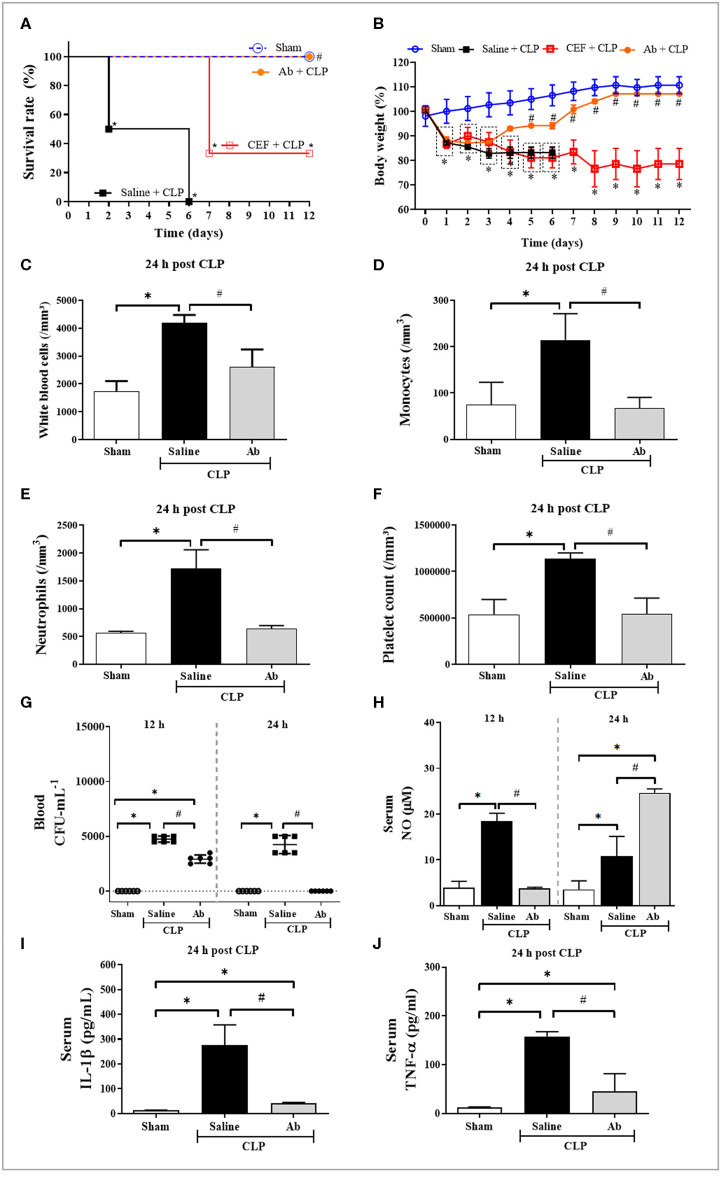
Effects of *A. brasiliensis* aqueous extract on survival rate (%), body weight, hematological[[Inline Image]] parameters, bacterial burden, and systemic inflammatory response in septic mice. **(A)** Survival of septic animals pretreated with Ab (135 mg/kg), CEF (20 mg/Kg), or saline (0.9%). **(B)** Body weight during 12 days. **(C)** Total leukocyte counts 24 h post CLP. **(D)** Monocyte counts 24 h post CLP. **(E)** Neutrophil counts 24 h post CLP. **(F)** Platelet counts 24 h post CLP. **(G)** Bacterial load in the blood 12 and 24 h post CLP. **(H)** NO levels 12 h and 24 h after CLP. **(I)** IL-1β and **(J)** TNF-α levels in serum of septic animals 24 h post CLP. Data presented as mean ± SD. (*p < 0.05 Saline-CLP or Ab-CLP vs. Sham; #*p* < 0.05 Ab-CLP vs. Saline-CLP).

As showed in Figure 2A, saline-pretreated septic mice (saline-CLP) died within six days, while 100% of Ab-pretreated septic animals (Ab-CLP) survived up to 12 days after CLP induction. Moreover, ceftriaxone-pretreated CLP group showed only 40% survival until 12th day. Regarding hematological parameters and inflammatory mediators, mice from saline-CLP group showed a significant augment in the number of circulating total leukocytes (Figure 2C), monocytes (Figure 2D), neutrophils (Figure 2E), and platelets (Figure 2F). Moreover, CLP increased the systemic levels of NO (Figure 2H), IL-1β (Figure 2I), and TNF-α (Figure 2J). On the other hand, Ab-CLP group maintained normal hematological parameters compared to sham group (Figures 2C–F). These animals produced low systemic levels of NO at 12 h of infection compared to saline-CLP group, increasing this production at 24 h post infection (Figure 2H). In addition, the pretreatment with Ab extract leads to a complete control of bacteremia 24 h post infection (Figure 2G), associated with an augment in NO (Figure 2H), and a significant decrease in IL-1β (Figure 2I) and TNF-α (Figure 2J) levels in septic animals.

### Aqueous Extract of *A. brasiliensis* Modulate Inflammatory Response and Increase Bacterial Killing

**Figure 3:**
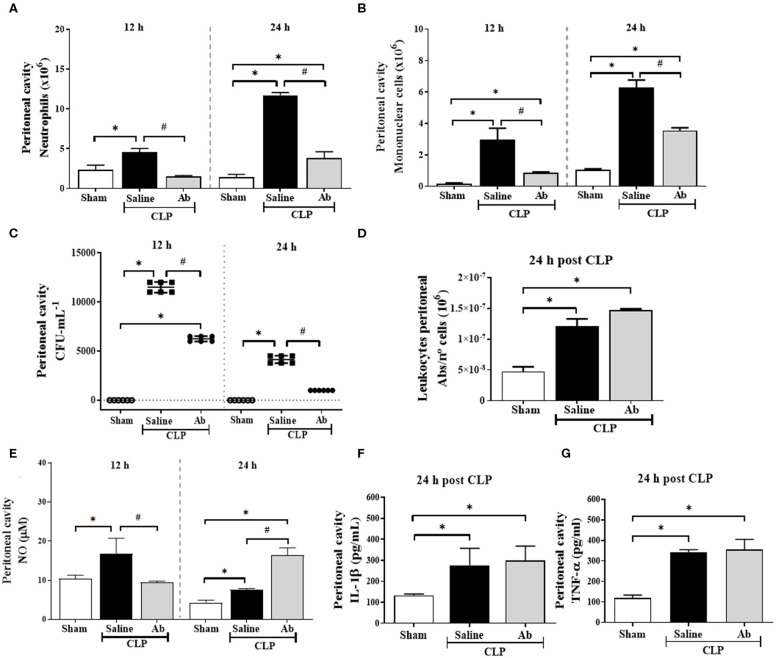
Effects of *A. brasiliensis* aqueous extract on microbicidal activity, cell migration, bacterial burden, and inflammatory mediators in the peritoneal cavity of septic mice. **(A)** Neutrophils, **(B)** mononuclear cells count and **(C)** CFU quantification in the peritoneal cavity of septic mice at 12 and 24 h post CLP. **(D)** *In vitro* phagocytic activity of zymosan particles by phagocytic peritoneal cells from septic animals after 24 h of CLP induction. **(E)** Nitric oxide, **(F)** IL-1β and **(G)** TNF-α levels in the peritoneal lavage fluid of septic mice. Data presented as mean ± SD. (*p < 0.05 Saline-CLP or Ab-CLP vs. Sham; #*p* < 0.05 Ab-CLP vs. saline-CLP).

Septic mice showed a significant increase in neutrophil and mononuclear cell recruitment to the peritoneal cavity at 12 h after sepsis induction (Figures 3A,B), as well as high bacterial load (Figure 3C) and increased NO levels (Figure 3E). In addition, at 24 h, these animals presented significant amount of NO (Figure 3E), IL-1β (Figure 3F), and TNF-α (Figure 3G) into peritoneal fluid and recruited cells showed increased phagocytic ability compared to that from sham group (Figure 3D). However, Ab-CLP group, at 12 h, showed a significant reduction in the influx of neutrophils and mononuclear cells (Figures 3A,B) and in NO production (Figure 3E) compared to saline-CLP animals. On the other hand, at 24 h post CLP, the Ab-CLP group showed an increase in NO production in the peritoneal fluid (Figure 3E), without alteration in phagocytosis (Figure 3D), IL-1β (Figure 3F), and TNF-α (Figure 3G), accompanied by inhibition of bacterial load (Figure 3C).

### *A. brasiliensis* Increases the Antioxidant Status of Septic Mice

**Figure 4:**
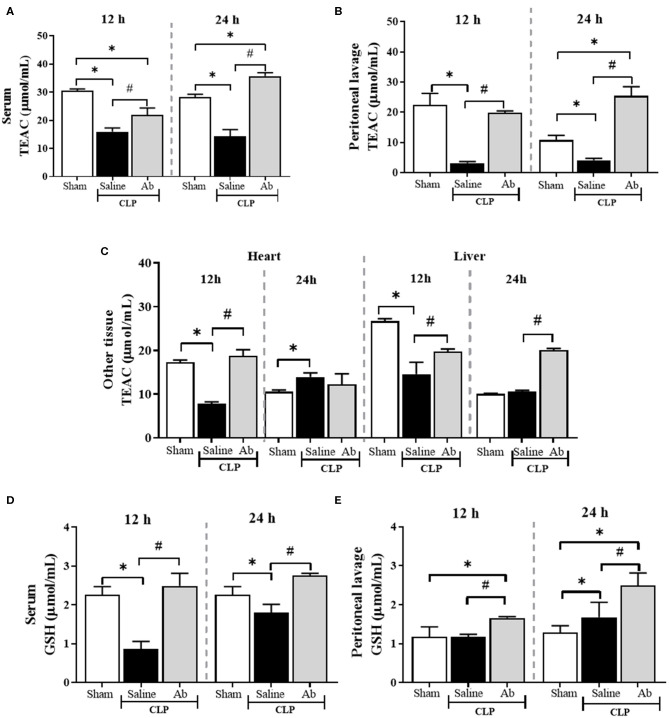
Effects of aqueous extract of *A. brasiliensis* on antioxidant parameters of septic mice 12 and 24 h after CLP. **(A)** TEAC in serum. **(B)** TEAC in the peritoneal lavage fluid. **(C)** TEAC levels in heart and liver. **(D)** GSH levels in serum. **(E)** GSH levels in peritoneal lavage fluid. The results were expressed as the mean ± SD. (*p < 0.05 Saline-CLP or Ab-CLP vs. Sham; #*p* < 0.05 Ab-CLP vs. saline-CLP).

In general, the antioxidant defense state was lower in septic animals compared to control animals (Figures 4A–D). Twelve hours post CLP, TEAC levels were reduced in serum, peritoneal cavity, heart and liver of septic mice treated with saline. Regarding antioxidant capacity based on GSH, saline-CLP animals showed a decrease in serum GSH levels at 12 and 24 h (Figure 4D). On the other hand, the pretreatment with Ab extract was able to improve the antioxidant defense state of septic animals in all tissues at 12 and 24 h post CLP (Figures 4A–E), with exception of heart at 24 h.

### Aqueous Extract of *A. brasiliensis* Reduce Oxidative Stress Markers and DNA Damage in Septic Animals

**Figure 5:**
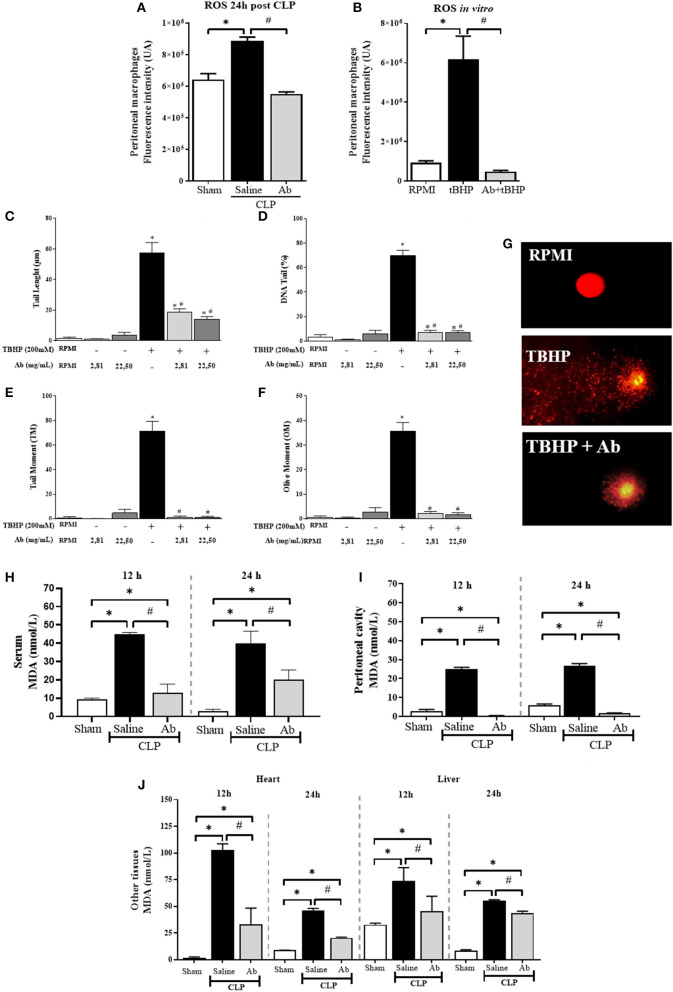
Effects of aqueous extract of *A. brasiliensis* on oxidative and genotoxic parameters of septic mice. **(A)** ROS production by peritoneal cells collected from septic animals 24 h after CLP induction. **(B)** ROS production by thioglycollate-elicited peritoneal macrophages after *in vitro* incubation with tBHP or tBHP plus Ab (22.5 mg/mL) **(C)** Tail length (μm), **(D)** DNA tail (%), **(E)** Tail Moment (TM), **(F)** Olive Moment (OM) in peripheral blood mononuclear cells from healthy volunteers incubated *in vitro* with tBHP or tBHP plus *A. brasiliensis*. **(G)** Representative images of comet assay of human cells incubated in RPMI medium, RPMI plus tBHP and tBHP plus Ab, **(H)** MDA levels in serum, **(I)** MDA levels in peritoneal cavity, and **(J)** MDA levels in heart and liver at 12 and 24 h after CLP. The results were expressed as the mean ± SD. (*p < 0.05 Saline-CLP or Ab-CLP vs. Sham; #*p* < 0.05 Ab-CLP vs. saline-CLP).

The *in vivo* treatment of septic mice with Ab caused significant inhibition in ROS production in response to infection (Figure 5A). In addition, Ab extract was also able to inhibit tBHP-induced ROS production *in vitro* by macrophages (Figure 5B), as well as tBHP-induced DNA damage in PBMC of health donors (Figures 5C–G). Regarding the most used lipid marker of oxidative stress, MDA levels were significantly increased in serum, peritoneal cavity, heart and liver of septic mice (Figures 5H–J). The pretreatment of animals with Ab caused strong decrease in MDA levels in all tissues evaluated, being completely inhibited in the peritoneal cavity, the main focus of bacteria (Figures 5H–J). These protective findings of *A. brasiliensis* were also demonstrated by the MDA/TEAC ratio (Table 1).

**Table 1:** MDA/TEAC ratios in samples from CLP-induced septic mice treated or not with *A. brasiliensis*.

**MDA/TEAC (mean ± SD)**					
**Time**	**Group**	**Serum**	**Peritoneal cavity**	**Heart**	**Liver**
12 h	Sham	0.30 ± 0.017	0.11 ± 0.001	0.07 ± 0.008	1.19 ± 0.026
	Saline-CLP	2.83 ± 0.18^a^	8.04 ± 1.135^a^	13.53 ± 0.178^a^	5.52 ± 0.416^a^
	Ab-CLP	0.51 ± 0.051^b^	0.02 ± 0.002^b^	2.15 ± 0.412^a, b^	1.94 ± 0.561^a, b^
24 h	Sham	0.08 ± 0.024	0.55 ± 0.017	0.83 ± 0.300	0.89 ± 0.013
	Saline-CLP	2.78 ± 0.004^a^	6.49 ± 0.833^a^	3.45 ± 0.120^a^	5.33 ± 0.076^a^
	Ab-CLP	0.50 ± 0.050^b^	0.05 ± 0.009^b^	1.66 ± 0.462^a, b^	2.20 ± 0.074^b^

a *p < 0.05 Saline-CLP or Ab-CLP vs. Sham*.

b *p < 0.05 Ab-CLP vs. Saline-CLP*.

## Discussion

In the present study, we showed for the first time the immunomodulatory and antioxidant protective effect of prophylactic *A. brasiliensis* aqueous extract treatment in the CLP-induced sepsis. Ab protected mice against sepsis by increasing bacterial clearance and survival, maintaining normal hematological parameters. The pretreatment with Ab reduced the systemic levels of inflammatory cytokines (TNF-α, IL-1β), increased the antioxidant response in several organs and tissues (GSH and TEAC) with concomitant inhibition of oxidative damage (lipid peroxidation in serum, peritoneal cavity, heart and liver) of septic mice.

Ab is a basidiomycete mushroom considered nutraceutical. Our group have reported that Ab has bioactive compounds such as phytosterols, aromatic amino acids, flavonoids and phenolic compounds (33). Phenolic compounds have a high antioxidant capacity according to their structure, depending on the number and position of the hydroxyl and act through enzymatic inhibition or in the trace elements sequestration, reducing reactive species formation (34). In addition, the aqueous extract of Ab is rich in polysaccharides such as β-glucans (11), which can activate leukocytes and increase phagocytosis and antimicrobial activity (35, 36).

In this study, Ab improved the survival of septic mice by an immunomodulatory mechanism. Our data are in agreement with previous reports showing that oral supplementation of Ab improved Crohn's disease through the reduction of systemic pro-inflammatory cytokines such as IL-1β, IL-6 and G-CSF. In addition, patients with ulcerative colitis presented decreased levels of IL-2, IL-5 and MIP-1β after 21 days of consumption compared placebo group (37). Moreover, the antitumor effect in multiple myeloma and cervical cancer mediated by an immunomodulatory activity have been reported (38, 39). These antimicrobial, antioxidant and immunomodulation properties described in preclinical and clinical studies with Ab supplementation may be associated with bioactive compounds found in mushroom, such as phenolic compounds, organic acids, amino acids and β-glucans (11, 12).

In sepsis, the pretreatment with antibiotics have a role to prevent complications such as systemic infections, reducing the mortality of patients (40). Here, the pretreatment with aqueous extract of Ab modulated the systemic and local release of proinflammatory cytokines, inhibited the leukocyte infiltration into peritoneal cavity, increased phagocytosis and NO production in infectious focus, leading to complete inhibition of bacterial burden in blood and peritoneal cavity. Smiderle et al. showed that the expression of proinflammatory cytokines (TNF-α and IL-1β) in LPS-stimulated THP-1 macrophages were inhibited by *in vitro* incubation with Ab-isolated β-glucans in presence of LPS (35). In addition, it was showed that the β-glucans negatively downregulated TLR-2 and TLR-4 receptors, decreasing exacerbated systemic immune system activation (41). It is important to point out that until now, there are no studies with *A. braziliensis* aqueous extract or any isolated compound in sepsis model.

In relation to the primary site of infection, Ab treatment modulated the inflammatory response into peritoneal cavity of septic mice, inducing almost complete bacterial burden elimination 24 h post CLP and a significant augment in NO levels, without alterations in IL-1β and TNF-α levels in relation to CLP-saline treated mice. In agreement, it was demonstrated that (16)-β-D-glucans activates dectin-1 receptors in monocytes, neutrophils and dendritic cells leading to phosphorylation of Syk and activation of CARD9/Bcl10/MALT-1 4 with consequent augment in phagocytic capacity and increased generation of ROS and RNS (42). Furthermore, according to Vitak et al., Ab is rich in arginine, a precursor to nitric oxide production by NO synthases responsible for the conversion of L-arginine to NO and L-citrulline (43).

Studies have shown that NO has dual effect during sepsis. NO contributes to elimination of pathogens through DNA nitrosation and desamination and inhibiting the action of bacterial DNA repair enzymes at the site of infection (44). On the other hand, studies have shown that NO modulates the expression of adhesion molecules and reduces leukocyte recruitment which can lead to microvascular dysfunction (45). However, although pretreatment with Ab reduced migration of leukocytes we observed an increase in NO levels and decreased bacterial burden. This may be due to the immunomodulatory property of Ab which might be correlated with increased phagocytic capacity and mechanisms reported above (44).

In addition, in sepsis the uncontrolled inflammatory response can cause oxidative stress, that plays a critical role in the pathogenesis and dysfunctions in multiple organs (46). In this respect our data showed that macrophages from septic animals produce higher levels of ROS compared to cells from sham or *Ab* treated animals. Moreover, *Ab* was able to inhibit the production of ROS by macrophages stimulated *in vitro* with tBHP, and also tBHP-induced DNA damage in human PBMC. In this context, Angeli et al. reported that the pretreatment with β-glucans extracted from Ab presented protective effect in human hepatic cells against the genotoxic and mutagenic effects of carcinogenic compound (Benzo[a]Pyrene) (47).

In septic patients, MDA levels are elevated and may be correlated with clinical worsening (48, 49). In our study, the levels of lipid peroxidation were significantly decreased in serum, peritoneal cavity, heart and liver of septic animals treated with Ab. These results are in agreement with evidences showing that the association of oxidative stress with systemic abnormalities in microcirculatory blood flow lead to cardiovascular and hepatic changes, that contributes to the high mortality of patients (50). In fact, Yan et al. reported that in CLP model, liver tissue damage starts ~1 h after sepsis, while cardiac dysfunction starts at 6 h (50).

In relation to the antioxidant defense, decreased levels of GSH and the inhibition of total antioxidant capacity are associated with organ failure and high mortality in sepsis (51). In contrast to septic animals, *Ab*-treated animals showed increased TEAC and GSH levels in serum and peritoneal cavity, and also enhanced antioxidant capacity in heart in liver tissues, as well as increased MDA/TEAC ratios in serum, peritoneal cavity, heart and liver, suggesting that the protective effect of *Ab* in CLP model is at least in part due to its antioxidant property.

Recently, De Souza et al. showed that the treatment of adjuvant-induced arthritic rats with Ab aqueous extract, at dose of 400 mg/kg, cause significant decrease in lipid damage in liver, brain, and plasma of treated rats. Moreover, the extract maintained the antioxidant defense, preserving the levels of reduced glutathione and protein thiol (52). In this line, it was demonstrated that in diabetic rats, the administration of Ab restored superoxide dismutase, catalase, and gluthatione peroxidase activity (43). This ability may be, at least in part, due to the presence of glutamic acid and glycine in Ab, since these amino acids are precursors of GSH synthesis, where glutamic acid reacts with cysteine to produce γ-glutamylcysteine (GCS) and subsequently reacts with glycine to form GSH (11, 53).

## Conclusion

**Figure 6:**
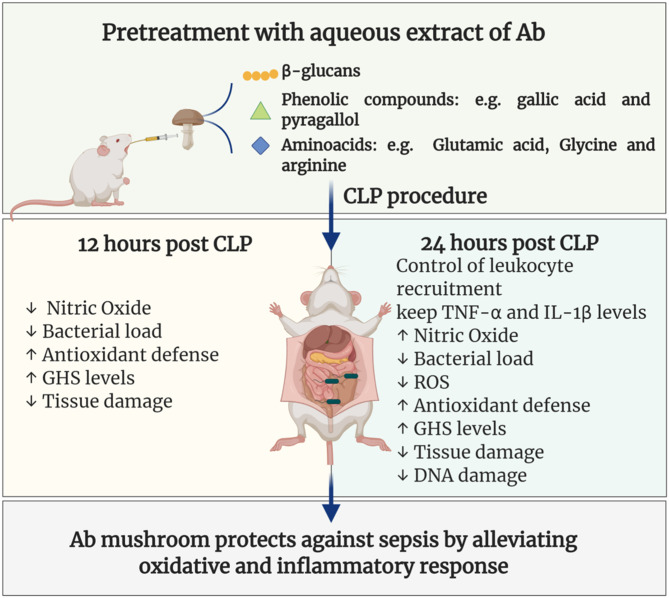
Aqueous extract of *A. brasiliensis* enhanced survival and reduced organ damage and oxidative stress in CLP sepsis model. Pretreatment with Ab aqueous extract in CLP septic mice was able to increase survival, reduce bacterial load, control leukocyte recruitment, reduce inflammatory cytokines, oxidative stress markers, and tissue damage, as well as, increases antioxidant defense.

In conclusion, it was showed for the first time that the pretreatment with *Ab* aqueous extract was able to increase the survival of septic animals by a mechanism involving immunomodulatory and antioxidant protective effects as summarized in Figure 6. Further studies are needed to better elucidate the immunomodulatory mechanisms and ensure the safety of their clinical use.

## Data Availability Statement

The datasets generated for this study are available on request to the corresponding author.

## Ethics Statement

This study was carried out in strict accordance with the recommendations of the Guide for the Care and Use of Laboratory Animals of the Brazilian National Council of Animal Experimentation (http://www.sbcal.org.br/) and the NIH Guidelines for the Care and Use of Laboratory Animals. The institutional Committee for Animal Ethics of Federal University of Pará/UFPA (CEUA, Protocol: 02/15) approved all the procedures used in this study. The *in vitro* tests, human venous blood was collected from healthy volunteers signing the Informed Consent Form (ICF). This study was approved by the Institutional Committee of Ethics in Research involving human beings from the health sciences sector of UFPA (CEP-ICS/UFPA), under n° 3544380 and CAAE 12776619.0.0000.0018.

## Author Contributions

KN-L and MM designed the study. KN-L, VM, AO, SF, and JO conducted most of the experiments and wrote the main manuscript. HS and VS donated *Agaricus brasiliensis*. CR and JR conducted oxidative stress assay. RS conducted most of the experiments. AP and PR performed quantification of cytokines corrected the manuscript. All authors read and approved the final manuscript.

## Conflict of Interest

The authors declare that the research was conducted in the absence of any commercial or financial relationships that could be construed as a potential conflict of interest.

## Abbreviations

Ab: *Agaricus brasiliensis*
ABTS: 2,2′-Azino-bis (3-ethylbenzothiazoline-6-sulfonic acid)
ANOVA: Analysis of Variance
Bcl10: B-Cell Lymphoma/Leukemia 10
CARD9: Caspase Recruitment Domain-Containing Protein 9
CEF: Ceftriaxone
CFU: Colony-Forming Units
CLP: Cecal Ligation and Puncture
DCF: Dichloro-Fluorescein
DMSO: Dimethylsulfoxide
DTNB: 5,5′-Dithiobis (2-nitrobenzoic acid)
EDTA: Ethylenediamine Tetraacetic Acid
ELISA: Enzime-Linked Immunosorbent Assay
FBS: Fetal Bovine Serum
GCS: Glutamylcysteine Synthase
*G*-*CSF*: Granulocyte Colony-Stimulating Factor
GSH: Glutathione
H_2_DCF-DA: Dichlorodihydrofluorescein Diacetate
HEPES: 4-(2-Hydroxyethyl)-1-Piperazineethanesulfonic Acid
ICF: Informed Consent Form
IL: Interleukin
LPS: Lipopolysaccharide
MALT1: Mucosa-Associated Lymphoid Tissue Lymphoma Translocation Protein 1
MDA: Malondialdehyde
MIP-1β: Macrophage Inflammatory Protein 1β
NF-κB: Nuclear Factor Kappa B
NO: Nitric Oxide
NO_2_: Nitrite
OM: Olive Moment
PBMC: Peripheral Blood Mononuclear Cells
PBS: Phosphate-Buffered Saline
PPARα: Peroxisome *Proliferator-Activated Receptor* alpha
RNS: Reactive Nitrogen Species
ROS: Reactive Oxygen Species
RPMI: Roswell Park Memorial Institute
TAS: Total Antioxidant Status
TBA: Thiobarbituric Acid
TBARS: Thiobarbituric Acid-Reactive Substances
tBHP: *tert*-Butyl Hydroperoxide
TCA: Trichloroacetic Acid
TEAC: Trolox Equivalent Antioxidant Capacity
TL: Tail Length
TLR: Toll-Like Receptor
TM: Tail Moment
TNF-α: Tumor Necrosis Factor Alpha.

## References

[1] World Health Organization Sepsis. (2018). Available online at: https://www.who.int/news-room/fact-sheets/detail/sepsis (Accessed March 01, 2020).

[2] Barros LLS Maia CSF Monteiro MC Fatores de risco associados ao agravamento de sepse em pacientes em unidade de terapia intensiva. Cad saúde colet. (2016) 24:388–96. 10.1590/1414-462x201600040091

[3] Vincent JL Jones G David S Olariu E Cadwell KK. Frequency and mortality of septic shock in europe and north america: a systematic review and meta-analysis. Crit Care. (2019) 23:196. 10.1186/s13054-019-2478-6 31151462 PMC6545004

[4] Gül F Arslantaş MK Cinel I Kumar A. Changing definitions of sepsis. Turk J Anaesthesiol Reanim. (2017) 45:129–38. 10.5152/TJAR.2017.93753 28752002 PMC5512390

[5] Nagar H Piao S Kim CS. Role of mitochondrial oxidative stress in sepsis. Acute Crit Care. (2018) 33:65–72. 10.4266/acc.2018.00157 31723865 PMC6849061

[6] Kumar S Gupta E Srivastava VK Kaushik S Saxena J Goyal LK. Nitrosative stress and cytokines are linked with the severity of sepsis and organ dysfunction. Br J Biomed Sci. (2019) 76:29–34. 10.1080/09674845.2018.1543160 30379116

[7] Prauchner CA. Oxidative stress in sepsis: pathophysiological implications justifying antioxidant co-therapy. Burns. (2017) 43:471–85. 10.1016/j.burns.2016.09.023 28034666

[8] Ruiz S Vardon-Bounes F Merlet-Dupuy V Conil JM Buléon M Fourcade O. Sepsis modeling in mice: ligation length is a major severity factor in cecal ligation and puncture. Intensive Care Med Exp. (2016) 4:22. 10.1186/s40635-016-0096-z 27430881 PMC4949182

[9] de Souza Gomes R Navegantes-Lima KC Monteiro VVS de Brito Oliveira AL Rodrigues DVS Reis JF Salivary gland extract from *aedes aegypti* improves survival in murine polymicrobial sepsis through oxidative mechanisms. Cells. (2018) 7:182 10.3390/cells7110182 PMC6262460 30360497

[10] Carvajal AESS Koehnlein EA Soares AA Eler GJ Nakashima ATA Bracht A Bioactives of fruiting bodies and submerged culture mycelia of *Agaricus brasiliensis* (*A. blazei*) and their antioxidant properties. Food Sci Technol. (2012) 46:493–9. 10.1016/j.lwt.2011.11.018

[11] Cho SM Jang KY Park HJ Park JS. Analysis of the chemical constituents of *Agaricus brasiliensis*. Mycobiology. (2008) 36:50-54. 10.4489/MYCO.2008.36.1.050 23997608 PMC3755252

[12] da Silva de Souza AC Correa VG Goncalves GA Soares AA Bracht A Peralta RM. Agaricus blazei bioactive compounds and their effects on human health: benefits and controversies. Curr Pharm Des. (2017) 23:2807–34. 10.2174/1381612823666170119093719 28103773

[13] Navegantes KC Albuquerque RFV Dalla-Santa HS Soccol CR Monteiro MC *Agaricus brasiliensis* mycelium and its polysaccharide modulate the parameters of innate and adaptive immunity. Food Agricult Immunol. (2013) 24:393–408. 10.1080/09540105.2012.691089

[14] Rubel R Santa HSD Dos Santos LF Fernandes LC Figueiredo BC Soccol CR. Immunomodulatory and antitumoral properties of *ganoderma lucidum* and *Agaricus brasiliensis* (Agaricomycetes) medicinal mushrooms. Int J Med Mushrooms. (2018) 20:393–403. 10.1615/IntJMedMushrooms.2018025979 29953399

[15] Akramiene D Kondrotas A Didziapetriene J Kevelaitis E. Effects of beta-glucans on the immune system. Medicina (Kaunas). (2007) 43:597–606. 10.3390/medicina43080076 17895634

[16] Firenzuoli F Gori L Lombardo G. The medicinal mushroom *Agaricus blazei* murrill: review of literature and pharmaco-Toxicological problems. Evid Based Complement Alternat Med. (2008) 5:3–15. 10.1093/ecam/nem007 18317543 PMC2249742

[17] Yim HS Chye FY Tan CT Ng YC Ho CW. Antioxidant activities and total phenolic content of aqueous extract of *pleurotus ostreatu*s (cultivated oyster mushroom). Malays J Nutr. (2010) 16:281–91. 22691933

[18] Val CH Brant F Miranda AS Rodrigues FG Oliveira BCL Santos EA. Effect of mushroom *Agaricus blazei* on immune response and development of experimental cerebral malaria. Malar J. (2015) 14:1–13. 10.1186/s12936-015-0832-y 26260055 PMC4531523

[19] González-Palma I Escalona-Buendía HB Ponce-Alquicira E Téllez-Téllez M Gupta VK Díaz-Godínez G. Evaluation of the antioxidant activity of aqueous and methanol extracts of *pleurotus ostreatus* in different growth stages. Front Microbiol. (2016) 7:1099. 10.3389/fmicb.2016.01099 27462314 PMC4940407

[20] Dalla Santa HS Rubel R Vitola FMD Leifa F Tararthuch AL Lima Filho Cavalcante JH Kidney function indices in mice after long intake of *Agaricus brasiliensis* mycelia (*Agaricus blazei, Agaricus subrufescens*) produced by solid state cultivation. J Biol Sci. (2009) 9:21–8. 10.3844/ojbsci.2009.21.28

[21] Song T Yang M Chen J Huang L Yin H He T. Prognosis of sepsis induced by cecal ligation and puncture in mice improved by anti-Clonorchis *sinensis cyclopholin* a antibodies. Parasites Vec. (2015) 8:1–10. 10.1186/s13071-015-1111-z 26427806 PMC4591565

[22] D'Acampora AJ Locks G de F. Median lethal needle caliber in two models of experimental sepsis. Acta Cir Bras. (2014) 29:1–6. 10.1590/S0102-86502014000100001 24474171

[23] Granger DL Taintor RR Boockvar KS Hibbs JB. Measurement of nitrate and nitrite in biological samples using nitrate reductase and griess reaction. Methods Enzymol. (1996) 268:142–51. 10.1016/s0076-6879(96)68016-1 8782580

[24] Stuehr DJ Marletta M A. Mammalian nitrate biosynthesis: mouse macrophages produce nitrite and nitrate in response to *escherichia coli* lipopolysaccharide. Proc Natl Acad Sci USA. (1985) 82:7738–42. 10.1073/pnas.82.22.7738 3906650 PMC391409

[25] Yamazaki RK Bonatto SJR Folador A Pizatto N Oliveira HHP Vecchi R. Lifelong exposure to dietary fish oil alters macrophage responses in walker 256 tumor-bearing rats. Cell Immunol. (2004) 231:56–62. 10.1016/j.cellimm.2004.12.001 15919370

[26] Albuquerque R V Malcher NS Amado LL Coleman MD Dos Santos DC Borges RS. *In vitro* protective effect and antioxidant mechanism of resveratrol induced by dapsone hydroxylamine in human cells. PLoS ONE. (2015) 10:e134768. 10.1371/journal.pone.0134768 26284371 PMC4540410

[27] Kohn H Liversedge M On a new aerobic metabolite whose production by brain is inhibited by apomorphine, emetine, ergotamine, epinephrine, and menadione. J Pharmacol Experimen Ther Nov. (1944) 82:292–300.

[28] Percario S Vital A Jablonka F Dosagem do malondialdeido. Newslab. (1944) 2:46–50.

[29] Miller NJ Rice-evans C Davies M Gopinathan V Milner A. A novel method for measuring antioxidant capacity and its application to monitoring the antioxidant status in premature neonates. Clin Sci. (1993) 84:407–12. 10.1042/cs0840407 8482045

[30] Re R Pellegrini N Proteggente A Pannala A Yang M Rice-Evans C. Antioxidant activity applying an improved aBTS radical cation decolorization assay. Free Radic Biol Med. (1999) 26:1231–7. 10.1016/s0891-5849(98)00315-3.36 10381194

[31] Ellman GL. Tissue sulfhydryl groups. Arch Biochem Biophys. (1959) 82:70–7. 10.1016/0003-9861(59)90090-6 13650640

[32] Ferreira-Cravo M Piedras FR Moraes TB Ferreira JLR de Freitas DPS Machado MD. Antioxidant responses and reactive oxygen species generation in different body regions of the estuarine polychaeta *Laeonereis acuta* (Nereididae). Chemosphere. (2008) 66:1367–74. 10.1016/j.chemosphere.2006.06.050 16884763

[33] de Oliveira FM Mokochinski JB Reyes Torres Y Dalla Santa HS González-Borrero PP. Photoacoustic spectroscopy applied to the direct detection of bioactive compounds in *Agaricus brasiliensis* mycelium. J Biol Phys. (2018) 44:93-100. 10.1007/s10867-017-9478-z 29210029 PMC5835002

[34] Minatel IO Borges CV Ferreira MI Gomez HAG Chen C-YO Lima GPP Phenolic compounds: functional properties, impact of processing and bioavailability. Phenolic Compd - Biol Act. (2017) 1:1–24 10.5772/66368

[35] Smiderle FR Alquini G Tadra-Sfeir MZ Iacomini M Wichers HJ Van Griensven LJLD. *Agaricus bisporus* and *Agaricus brasiliensis* (1 → 6)-β-d-glucans show immunostimulatory activity on human THP-1 derived macrophages. Carbohydr Polym. (2013) 94:91–9. 10.1016/j.carbpol.2012.12.073 23544515

[36] Yamanaka D Tada R Adachi Y Ishibashi K Motoi M. *Agaricus brasiliensis*-derived β-glucans exert immunoenhancing effects via a dectin-1-dependent pathway. Int Immunopharmacol. Elsevier B.V. (2012) 14:311–9. 10.1016/j.intimp.2012.07.017 22878139

[37] Therkelsen SP Hetland G Lyberg T Lygren I Johnson E Effect of the medicinal *Agaricus blazei* murill-Based mushroom extract, andoSanTM, on symptoms, fatigue and quality of life in patients with crohn's disease in a randomized single-Blinded placebo controlled study. PLoS ONE. (2016) 11:e159288 10.1371/journal.pone.0159288 PMC4944955 27415795

[38] Tangen JM Tierens A Caers J Binsfeld M Olstad OK Trøseid A-MS. Immunomodulatory effects of the *Agaricus blazei* murrill-based mushroom extract andoSan in patients with multiple myeloma undergoing high dose chemotherapy and autologous stem cell transplantation: a randomized, double blinded clinical study. Biomed Res Int. (2015) 2015:1–11. 10.1155/2015/718539 25664323 PMC4312620

[39] Ahn W-S Kim D-J Chae G-T Lee J-M Bae S-M Sin J-I. Natural killer cell activity and quality of life were improved by consumption of a mushroom extract, *Agaricus blazei* murill kyowa, in gynecological cancer patients undergoing chemotherapy. Int J Gynecol Cancer. (2004) 14:589–94. 10.1111/j.1048-891X.2004.14403.x 15304151

[40] Mourad MM Evans R Kalidindi V Navaratnam R Dvorkin L Bramhall SR. Prophylactic antibiotics in acute pancreatitis: endless debate. Ann R Coll Surg Engl. (2017) 99:107–12. 10.1308/rcsann.2016.0355 27917667 PMC5392851

[41] Shah VB Williams DL Keshvara L. beta-Glucan attenuates tLR-2 and tLR4-mediated cytokine production by microglia. Neurosci Lett. (2009) 458:111–5. 10.1016/j.neulet.2009.04.039 19393720 PMC4435685

[42] Camilli G Tabouret G Quintin J. The complexity of fungal β-Glucan in health and disease: effects on the mononuclear phagocyte system. Front Immunol. (2018) 9:673. 10.3389/fimmu.2018.00673 29755450 PMC5932370

[43] Vitak TY Wasser SP Nevo E Sybirna NO Effect of medicinal mushrooms on l-arginine / nO system in red blood cells of streptozotocin-induced diabetic rats. Adv Diab Metab. (2016) 4:25–31. 10.13189/adm.2016.040201

[44] Kutty SK Ho KKK Kumars N Nitric oxide donors as antimicrobial agents. In: Seabra AB, editor. *Nitric Oxide Donors* Cambridge: Elsevier (2017). p. 169–89. 10.1016/B978-0-12-809275-0.00007-7

[45] Benjamim CF Silva JS Fortes ZB Oliveira MA Ferreira SH Cunha FQ. Inhibition of leukocyte rolling by nitric oxide during sepsis leads to reduced migration of active microbicidal neutrophils. Infect Immun. (2002) 70:3602–10. 10.1128/IAI.70.7.3602-3610.2002 12065501 PMC128083

[46] Luan YY Dong N Xie M Xiao XZ Yao YM. The significance and regulatory mechanisms of innate immune cells in the development of sepsis. J Interf Cytokine Res. (2014) 34:2–15. 10.1089/jir.2013.0042 24006870 PMC3887438

[47] Angeli JPF Ribeiro LR Bellini MF Mantovani MS. Beta-Glucan extracted from the medicinal mushroom *Agaricus blazei* prevents the genotoxic effects of benzo[a]pyrene in the human hepatoma cell line hepG2. Arch Toxicol. (2009) 83:81–6. 10.1007/s00204-008-0319-5 18528685

[48] Weiss SL Deutschman CS. Elevated malondialdehyde levels in sepsis - something to “stress” about? Crit Care. (2014) 18:125. 10.1186/cc13786 25029036 PMC4056888

[49] Lorente L Martín MM Abreu-González P Domínguez-Rodríguez A Labarta L Díaz C. Prognostic value of malondialdehyde serum levels in severe sepsis: a multicenter study. PLoS ONE. (2013) 8:e53741. 10.1371/journal.pone.0053741 23341989 PMC3544841

[50] Yan J Li S Li S. The role of the liver in sepsis. Int Rev Immunol. (2014) 33:498–510. 10.3109/08830185.2014.889129 24611785 PMC4160418

[51] Kim JS Kwon WY Suh GJ Kim KS Jung YS Kim SH. Plasma glutathione reductase activity and prognosis of septic shock. J Surg Res. (2016) 200:298–307. 10.1016/j.jss.2015.07.044 26316444

[52] de Souza ACDS Gonalves GA Soares AA de Sá-Nakanishi AB de Santi-Rampazzo AP Natali MRM Antioxidant action of an aqueous extract of royal sun medicinal mushroom, *Agaricus brasiliensis* (Agaricomycetes), in rats with adjuvant-Induced arthritis. Int J Med Mushrooms. (2018) 20:101–17. 10.1615/IntJMedMushrooms.2018025309 29773003

[53] Salyha NO Effects of l-glutamic acid and pyridoxine on glutathione depletion and lipid peroxidation generated by epinephrine-induced stress in rats. Ukr.Biochem.J. (2018) 90:102–10. 10.15407/ubj90.04.102

